# Atrial cardiomyopathy: Current and future imaging methods for assessment of atrial structure and function

**DOI:** 10.3389/fcvm.2023.1099625

**Published:** 2023-03-31

**Authors:** Cassia Kessler Iglesias, Jim Pouliopoulos, Liza Thomas, Christopher S. Hayward, Andrew Jabbour, Diane Fatkin

**Affiliations:** ^1^ Department of Cardiology, St Vincent's Hospital, Sydney, NSW, Australia; ^2^ Victor Chang Cardiac Research Institute, Sydney, NSW, Australia; ^3^ School of Clinical Medicine, Faculty of Medicine and Health, University of New South Wales, Sydney, NSW, Australia; ^4^ Westmead Clinical School, University of Sydney, Sydney, NSW, Australia; ^5^ Department of Cardiology Westmead Hospital, Sydney, NSW, Australia; ^6^ South West Clinical School, UNSW Sydney, Sydney, NSW, Australia

**Keywords:** atrial cardiomyopathy, atrial function, echocardiography, cardiac magnetic resonance (CMR), cardiac imaging

## Abstract

Changes in atrial size and function have historically been considered a surrogate marker of ventricular dysfunction. However, it is now recognized that atrial cardiomyopathy (ACM) may also occur as a primary myocardial disorder. Emerging evidence that ACM is a major risk factor for atrial fibrillation, heart failure, and thromboembolic stroke, has highlighted the significance of this disorder and the need for better assessment of atrial metrics in clinical practice. Key barriers in this regard include a lack of standardized criteria or hierarchy for the diagnosis of ACM and lack of consensus for the most accurate phenotyping methods. In this article we review existing literature on ACM, with a focus on current and future non-invasive imaging methods for detecting abnormalities of atrial structure and function. We discuss the relative advantages and disadvantages of transthoracic echocardiography and cardiac magnetic resonance imaging for assessing a range of parameters, including atrial size and contractile function, strain, tissue characteristics, and epicardial adipose tissue. We will also present the potential application of novel imaging methods such as sphericity index and four- or five-dimensional flow.

## 1. Introduction

**Figure 1:**
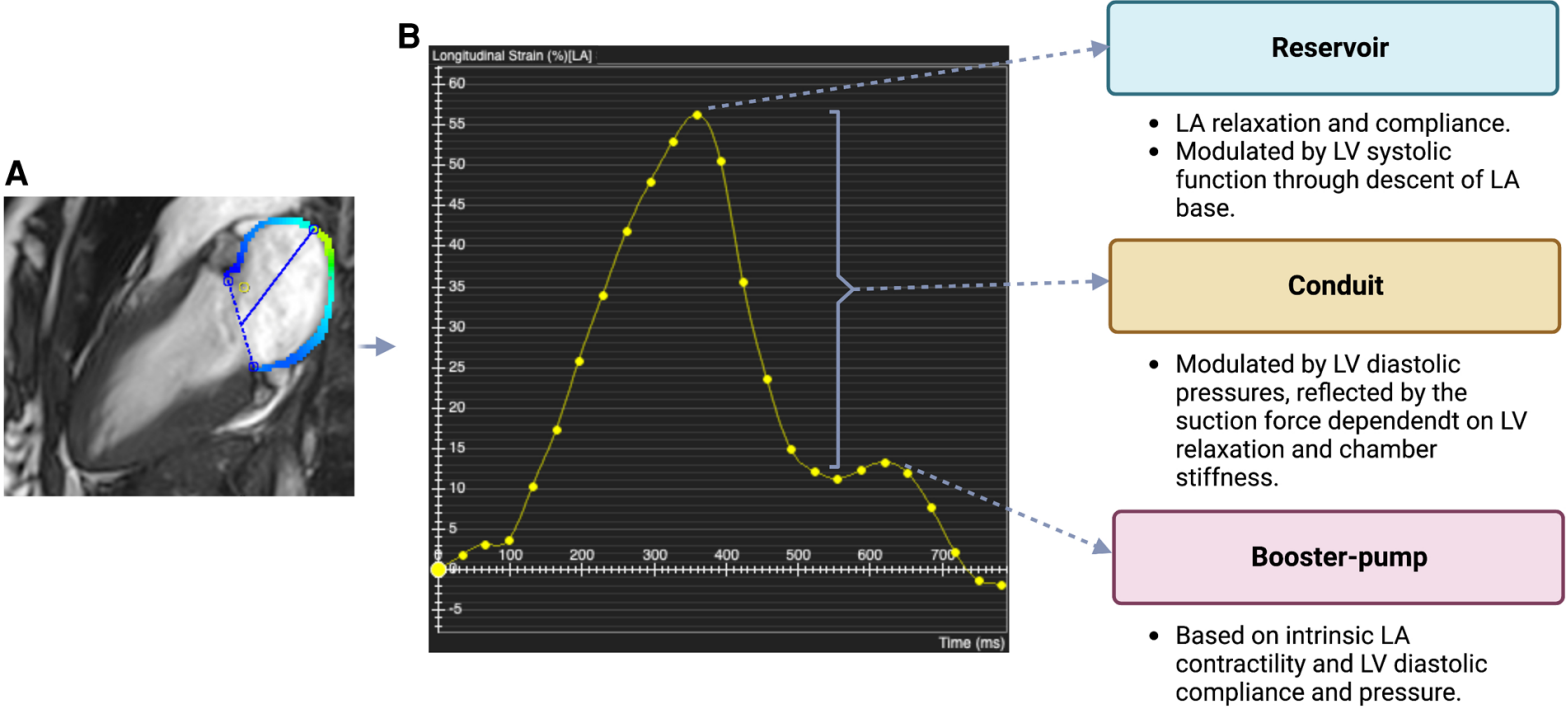
Phases of Atrial Function (**A**) cardiac magnetic resonance (CMR) long-axis 2-chamber view. Colored line (blue/green) represents the myocardial border of the left atrium (LA) where strain is measured. Dashed line and straight line mark LA width and length, respectively. The CMR image was acquired using a 3 T CMR scanner from our institute (St Vincent's Hospital Sydney, Australia) with ethics approval. (**B**) Line graph plotting global longitudinal LA strain (%) in 25 different points of the cardiac cycle (yellow dots). Reservoir strain = maximal value of the first (diastolic) peak; booster-pump strain = maximal value of the second (systolic) peak; conduit strain = difference between reservoir strain and booster-pump strain. LA and left ventricular (LV) factors that contribute to strain in each of these phases are indicated.

The left atrium (LA) plays an important role in cardiac performance by modulating left ventricular (LV) filling. There are 3 main functional phases (Figure 1), with the LA serving as a reservoir in systole, a conduit in early diastole, and booster pump in late diastole. There is interaction between the LA and LV in each phase. LA reservoir function represents LA relaxation and compliance, modulated by LV systolic function through descent of the mitral annulus and LA base, as well as LA filling pressure. LA conduit function relies on LV compliance and diastolic pressures reflected by the suction force dependent on active LV relaxation and chamber stiffness. LA booster pump function is based on intrinsic LA contractility and LV end-diastolic compliance and pressure (1). The LA acts as a volume sensor and barometer of LV diastolic function and has an essential role in communicating changes in mechanical stress with neurohormonal pathways (natriuretic peptides, sympathetic nervous system, renin-angiotensin-aldosterone system). LA size reflects the net effect of LV filling pressures over time, making it a useful index of both the chronicity and severity of LV diastolic dysfunction (2) as well as an early marker of ventricular disease (1).

LA enlargement has been widely recognized as prognostic marker of adverse cardiovascular events and all-cause mortality (3). LA dilatation has been associated with an increased risk of atrial fibrillation and stroke (4), overall mortality after myocardial infarction (5), death and hospitalization in patients with dilated cardiomyopathy (6), and major cardiac events or death in patients with diabetes mellitus (7). However, LA size reflects only one aspect of LA performance and does not capture the nuanced aspects of atrial function that are seen throughout the various phases of the cardiac cycle. Moreover, LA dysfunction may precede changes in LA size (1, 8). In recent years, there has been an increased emphasis on characterization of phasic LA function, i.e., reservoir, conduit, and booster function, in disease states and correlation of these parameters to adverse outcomes. As an example, LA contractile function has been proposed as a sensitive tool for detecting early stages of LV disease and atrial fibrillation (9), while LA reservoir strain demonstrated prognostic utility in heart failure with preserved ejection fraction (10) and chronic kidney disease (11).

Atrial cardiomyopathy (ACM) is an underdiagnosed and poorly studied condition that was recently defined by the European Heart Rhythm Association as: “Any complex of structural, architectural, contractile or electrophysiological changes affecting the atria with the potential to produce clinically relevant manifestations” (12). Within this framework, ACM was sub-classified on the basis of histological and pathophysiological characteristics into four classes: (i) principal cardiomyocyte changes, (ii) principally fibrotic changes, (iii) combined cardiomyocyte pathology and fibrosis, and (iv) primarily non-collagen infiltration (with or without cardiomyocyte changes) (12). Atrial failure, which can result from ACM, has been defined as: “Any atrial dysfunction (anatomical, mechanical, electrical, and/or rheological, including blood homeostasis) causing impaired heart performance and symptoms, and worsening quality of life or life expectancy, in the absence of significant valvular or ventricular abnormalities” (13). Both of these definitions are broad and potentially applicable to a myriad of defects and underlying causes. This has resulted in considerable confusion and the lack of precise criteria for diagnosing ACM in clinical settings.

## 2. Atrial imaging tools

Three non-invasive imaging techniques are most widely used for evaluating the atria: transthoracic echocardiography (TTE), cardiac magnetic resonance (CMR) and cardiac computed tomography (CT). Of these, TTE has historically been the most popular method. Despite advances in imaging techniques, TTE remains the modality of choice for screening and follow up, due to its widespread availability, ease and time of image acquisition, high temporal resolution, and relatively low cost. TTE is also safe, as there are no requirements for contrast agent or ionizing radiation. TTE is suited for measuring atrial size as well as phasic LA volumes. The main limitation of this technique is difficulty in gaining adequate acoustic windows in some patients, particularly in those who are obese. Since TTE has largely been a two-dimensional (2D) technique, another limitation when assessing chamber volumes and function is the need for geometric assumptions. Some of these limitations have been overcome with the emergence of three-dimensional (3D) echocardiography, which has automated border detection and good temporal resolution (Table 1) (2).

**Table 1:** Comparison of TTE and CMR for evaluation of atrial structure and function.

	TTE	CMR
Temporal resolution*	2D = 10–20 ms, 3D 50–75 ms, TDI = 5−10 ms, Speckle = 10–20 ms	25–50 ms
Spatial resolution*	2D = −0.5–1 mm 3D = 1–2 mm	1–2 mm
Limitation with imaging window	Yes	No
Availability	Wide	Limited
Cost	Low	High
Safety	Optional contrast	LGE and renal failure, claustrophobia, contraindication to older PPM and ICDs
Atrial Size and Volume	2D = Geometric limitations 3D = Good	Gold Standard
Atrial strain	Limited temporal and spatial resolution of strain analysis. Limitation with imaging window	Excellent visualization of LA wall. High special resolution to define endocardial borders
Tissue Characteristics	Limited	Gold standard Fibrosis, edema, intramyocardial fat
EAT	Limited to RV free wall	Whole heart EAT volume
Sphericity	2D = Limited by geometric assumptions 3D = Good	Accurate view of LA shape
Flow	Mitral valve, pulmonary veins and left atrial appendage only	Whole heart flow with 4D and 5D flow techniques

* Values given here are approximations from commonly used techniques described in literature (2); 2D = 2-dimensional; 3D = 3-dimensional; 4D = 4-dimensional; 5D = 5-dimensional; CMR = cardiac magnetic resonance; EAT = epicardial adipose tissue; LA = left atrium; RV = right ventricle; TDI = tissue Doppler imaging.

**Figure 2:**
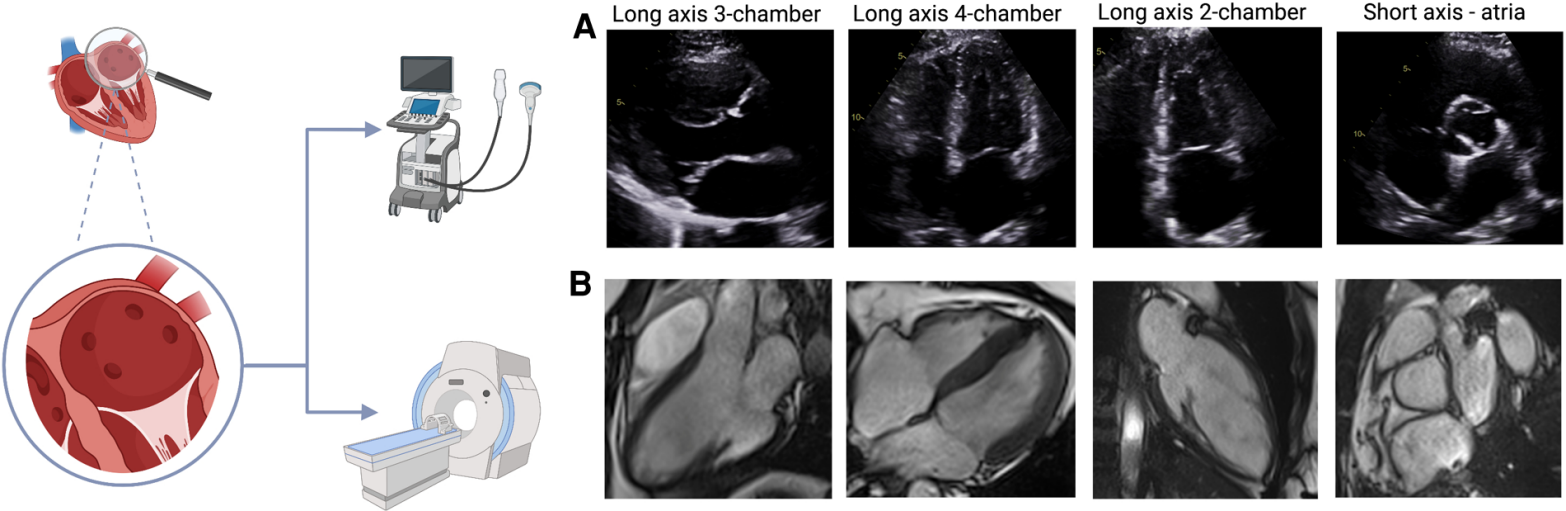
TTE and CMR views of the atria (**A**) TTE views of the atria: long axis 3-chamber, long axis 4-chamber, long axis 2-chamber, short axis. (**B**) CMR images of the atria in the same views. The images shown for each technique were acquired from our institute (St Vincent's Hospital Sydney, Australia) with ethics approval.

CMR is now considered the gold standard technique for non-invasive assessment of cardiac chamber volumes and function due to its excellent spatial and temporal resolution, reproducibility and accuracy, and ability to provide extensive and detailed tissue characterization. CMR overcomes the main problem of echocardiography, namely, limited acoustic windows, and hence reliably provides more accurate and high quality imaging data for all patients (Figure 2) (2). It is the most expensive of the three techniques and its availability in most centers is limited. The risks are low but not negligible and are related to the use of contrast agents such as gadolinium, when required, specifically in patients with renal failure.

Cardiac CT has been used in the literature for detection of LA appendage thrombus (14) and measurement of epicardial adipose tissue (EAT) (15). However, due to the need for radiation and contrast agent, its use solely for the purpose of assessing atrial structure cannot be recommended and it will not be discussed in this review paper.

In this review we will focus on non-invasive modalities that do not require ionizing radiation as these are preferable and more practical for potential use in routine evaluation of ACM in clinical practice.

### 2.1. Assessment of atrial size and phasic volumes

For many years, atrial size was estimated using TTE-based LA diameters from 2D images or M-mode tracings. This approach can lead to imprecise data if the orientation of the long axis of the LA is not optimal and true orthogonal diameters are unable to be obtained. Moreover, using data from a single linear dimension may not be a true representation of size if there are irregularities of atrial shape. LA dilatation often occurs in an asymmetric manner, particularly in medial-lateral and supero-inferior directions. Because of these issues, estimation of LA volume (LAV) has evolved as the preferred measurement of atrial size, with indexation for body mass. The European Society of Cardiology considers an indexed LAV (LAVi) above 34 ml/m^2^ as the threshold level for LA enlargement (16). Increases in LA size are usually attributed to increased wall tension due to LA pressure or volume load but may also reflect a primary atrial myocardial defect (12). Both TTE and CMR have been used to assess atrial reverse remodeling. Although there is no approved definition, it is considered that a 15% reduction of initial LAV mirrors the degree of reverse remodeling (17).

Due to geometric assumptions about LA shape and foreshortening of the LA cavity in the apical views (as the LA is a far field structure) LAV can be underestimated by TTE. Moreover, it has only been recently appreciated that the long axis of the LV is not the long axis of the LA and hence LAV may be underestimated by TTE if measured on a LV-focused image when compared with volumes obtained by 3D echocardiography and CMR (Figure 2) (16). TTE measures maximum LA volume at the time of mitral valve opening, but this only represents a snapshot of LA function at a specific point of the cardiac cycle. Assessment of LA phasic volumes has been proposed as a way to overcome this problem. LA phasic volumes include maximum LA volume (reservoir), pre-atrial contraction volume (conduit) and minimum LA volume (booster-pump). Total or global LA function can be measured by LA ejection fraction (LAEF), considering minimum and maximum LA volumes.

A number of novel echocardiography parameters have been derived for more detailed assessment of passive and active emptying volumes and fractions. This includes: global function, represented by total emptying fraction [(LAVmax—LAVmin)/LAVmax]; reservoir function, represented by LA expansion index [(LAVmax—LAVmin)/LAVmin)]; conduit function, represented by passive emptying fraction [LAVmax—LAVpreA)/LAVmax] and booster pump function, represented by the active emptying fraction [(LAVpreA- LAVmin)/LAVpreA] (1). The LA function index (LAFI) is an echocardiographic rhythm-independent measure of atrial function. The LAFI is a ratio that incorporates analogues of cardiac output, atrial reservoir function and LA size (18). Measurement of LA phasic volumes is time-consuming, and errors can arise from geometric assumptions of biplane volume calculations, as well as from difficulties with echocardiographic windows and the timing of various atrial events. 3D echocardiography for LAV can address some of these issues, but its use in clinical practice is still very limited.

In recent years CMR has been increasingly used to evaluate LA function (19) and normal ranges for LA phasic volumes and ejection fraction have been reported (20). Steady-state free precession (SSFP) cine sequences are typically used for atrial assessment, providing temporal resolution of around 25 to 50 ms. While this may not be as good as TTE, as a 3D modality without acoustic window limitation, CMR outperforms standard TTE for quantification of LA area and volume (21). Acquisition of multislice image stacks to include the LA is also used and overcomes the problem of geometrical assumptions that are encountered with TTE.

Atrial remodeling is initially adaptive, but when it occurs in response to a chronic pathological stimulus, it often becomes maladaptive and associated with an increased risk of adverse sequelae and mortality (22). Remodeling processes can be evidenced by changes in atrial size and function. LAVmax has been shown to be a biomarker for major cardiac events both in healthy individuals and in various cardiovascular conditions (23, 24), including myocardial infarction (5), heart failure (HF) (25), stroke (4), degenerative mitral regurgitation (26), and atrial fibrillation (AF) (27). Additionally, LA phasic volumes and function assessed by 3D TTE were strong predictors of cardiovascular events in an elderly population (28). LA size measured by CMR is reported as having an independent association with all-cause mortality (3), and LA function as an independent predictor of outcomes in patients with heart failure with preserved ejection fraction(HFpEF) (29).

### 2.2. Assessment of myocardial strain

In contrast to the LV, quantification of LA contraction is not readily performed using routine methods. Limited information about LA contractile function can be obtained from TTE using pulsed-wave Doppler measurements of LV diastolic filling and the peak velocities of mitral valve inflow E and A waves (30). In particular, the peak A wave velocity has been used as an indicator of atrial booster pump function. Mitral inflow patterns are influenced by age and loading conditions and thus do not necessarily equate precisely with intrinsic atrial function. Other TTE-based parameters that have been used to assess atrial function include PV atrial reversal velocity and Doppler tissue imaging-derived annular a' velocity (31). Assessment of myocardial strain has been used to evaluate LV function and is increasingly being used to evaluate LA phasic function. Strain and strain rate imaging provide data on myocardial deformation by estimating spatial gradients in myocardial velocities. Strain demonstrates the change of dimension relative to initial dimension, while strain rate is the instantaneous rate by which it occurs. LA mechanical dispersion, as defined by standard deviation of time to positive strain (SD-TPS), is a novel marker of atrial electromechanical function, and has been used for prediction of the new onset and recurrence of arrhythmias and risk of thrombus formation (32, 33).

2D speckle-tracking on TTE calculates strain by tracking tissue deformation frame-by-frame *via* characteristic myocardial speckles. It can be used as a more sensitive marker than ejection fraction to detect early functional remodeling before anatomical alterations occur (34). Unique challenges with strain imaging of the LA when compared to the LV include the thin LA wall, the complex LA motion during cardiac cycle, regional LA differences in contraction, and higher signal noise from surrounding structures. Another factor is the restricted field of view and signal attenuation due to the far field location of the atria with respect to the ventricles.

CMR-derived myocardial feature tracking (Figure 1) is a technique analogous to TTE-based speckle tracking, deriving quantitative deformation parameters from routinely available SSFP cine sequences. CMR provides an excellent visualization of the LA wall, with high spatial resolution and ability to define endocardial borders. CMR also provides an accurate view of the LA shape as a non-symmetrical 3D structure, without geometric assumptions. Methods to measure LA longitudinal strain using feature tracking algorithms for standard CMR SSFP cine images have recently gained importance (35). Studies comparing strain measured by different methods demonstrated better feasibility with CMR, as not all patients were found to have good tracking with TTE, albeit with lower temporal resolution (36).

In clinical practice, LA strain has been shown to detect subclinical changes associated with aging, gender and ethnicity (37, 38). LA strain is an emerging tool in the early diagnosis of heart disease associated with hypertension, diabetes (39), and HFpEF (40). LA strain has been reported to be a stronger predictor of cardiovascular outcomes than LA size alone (41), and is associated with recurrence of AF after ablation (42, 43) and aortic valve surgery (44).

### 2.3. Tissue characteristics

A major advantage of CMR is its ability to evaluate myocardial tissue characteristics. The presence and quantity of myocardial edema, interstitial fibrosis, and fat can be assessed using multiparametric mapping techniques and provide clues to underlying disease pathologies or prognosis (45).

**Figure 3:**
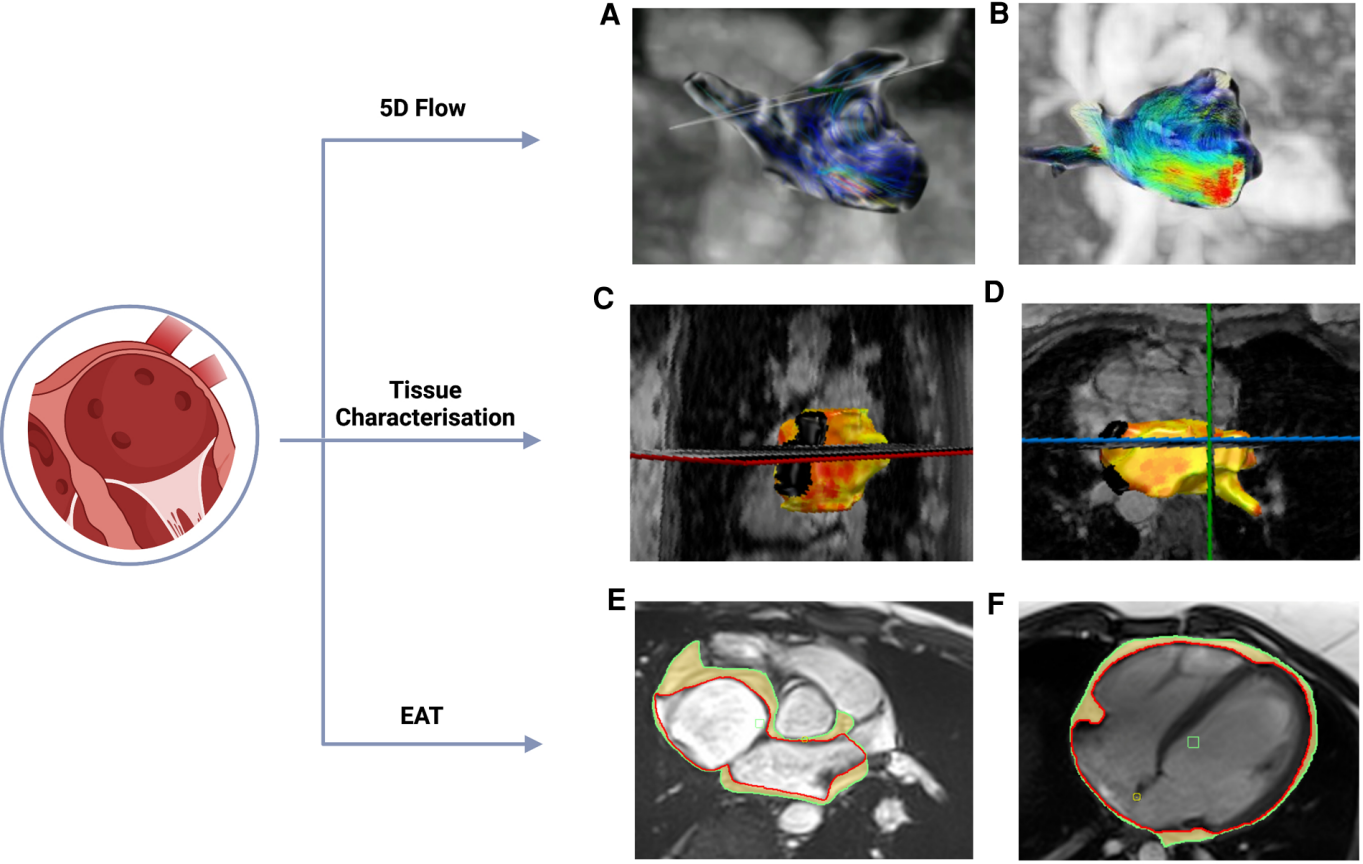
Novel CMR methods (**A&B**) 5-dimensional (5D) flow CMR: segmented left atrial view with flow direction represented by thin lines and flow velocities coded from high (red) to low (blue). (**C&D**) Detection of atrial fibrosis using CemrgApp (47). Atrial fibrosis in the long axis 2-chamber (**C**) and axial (**D**) views was quantified using 3D magnetic resonance angiography and 3D late gadolinium enhancement images. Fibrotic tissue (yellow/orange) is identified from elements of the surface of the atrial wall that have a fibrotic score above a given threshold. (**E&F**) Atrial EAT was assessed using CMR SSFP cine images in the axial (**E**) and long axis 4-chamber view. Red line marks the epicardium, green line marks the pericardium and highlighted in yellow is the EAT. The images were acquired using a 3 T CMR scanner from our institute (St Vincent's Hospital Sydney, Australia) with ethics approval.

There has been significant improvement in analysis techniques over the past few years but myocardial tissue characterization is not yet clinically available for the atria (46). Assessment of atrial fibrosis from CMR images (Figure 3) can be performed using late gadolinium enhancement (LGE) images with a high intra- and inter-observer correlation. Furthermore, the total volume of fibrotic tissue can be calculated as a percentage of the LA wall volume and subsequently categorized into stages (47, 48). The requirement for gadolinium-based contrast agents may be a contraindication to CMR atrial fibrosis assessment in some patients. Although quantification of atrial fibrosis has been demonstrated to be possible, there is still significant discrepancy in the amount of LA fibrosis detected between methods (49, 50). Even though CMR has an excellent spatial resolution to detect endocardial atrial wall borders, its ability to differentiate between the atrial myocardial and epicardial walls remains limited since the imaging signal of the LA wall can be subject to partial volume effect, in view of the thin atrial wall relative to the LGE-CMR voxel size (50).

Both atrial contractile function and fibrosis are associated with increased risk of AF (51). There is discrepancy in evidence for whether ablation treatment of AF with AF ablation should be guided by the presence and/or distribution of fibrotic areas identified by LGE-CMR. In a recent multi-center prospective trial, Delayed-Enhancement MRI Determinant of Successful Radiofrequency Catheter Ablation of Atrial Fibrillation (DECAAF I), there was a significant association between the burden of atrial fibrosis and arrhythmia recurrence after ablation procedures for atrial fibrillation (52). On the other hand, a second study published by the same group (DECAAF II) failed to demonstrate that CMR-guided fibrosis ablation plus pulmonary vein isolation (PVI) is more efficient in preventing arrhythmia recurrence than PVI catheter ablation alone (53). The presence of atrial fibrosis in patients with AF has also been associated with thromboembolic events (48). Atrial structural remodeling is present in patients without a history of AF and has been reported in hypertension, heart failure and valvular heart disease (54).

The assessment of atrial tissue characteristics should not be limited to fibrosis, since different types of fibrosis can co-exist in the atrial tissue, including interstitial and replacement fibrosis. The contribution of each type of fibrosis represented by CMR-LGE in the pathogenesis of AF is still unknown (53). In ventricular myocardium, CMR has been used to detect and quantify myocardial edema and intramyocardial fat. Nevertheless, these features have not yet been reported in the thin-walled atria. Other tissue characteristics that both CMR and Cardiac CT can detect include the total burden and distribution of EAT deposition, as well as myocardial infiltration as observed in cardiac amyloidosis.

### 2.4. Epicardial adipose tissue

Adipose tissue deposition is defined as “epicardial” when present between epicardium and pericardium, or “pericardial” if external to the pericardium. There are accumulating data to show that EAT is associated with the presence, severity, and recurrence of atrial fibrillation (55). The basis for this association is incompletely understood but is thought to be mediated by adipokines, inflammatory cytokines and other substances produced by EAT that exert paracrine effects on the adjacent myocardium (56).

**Figure 4:**
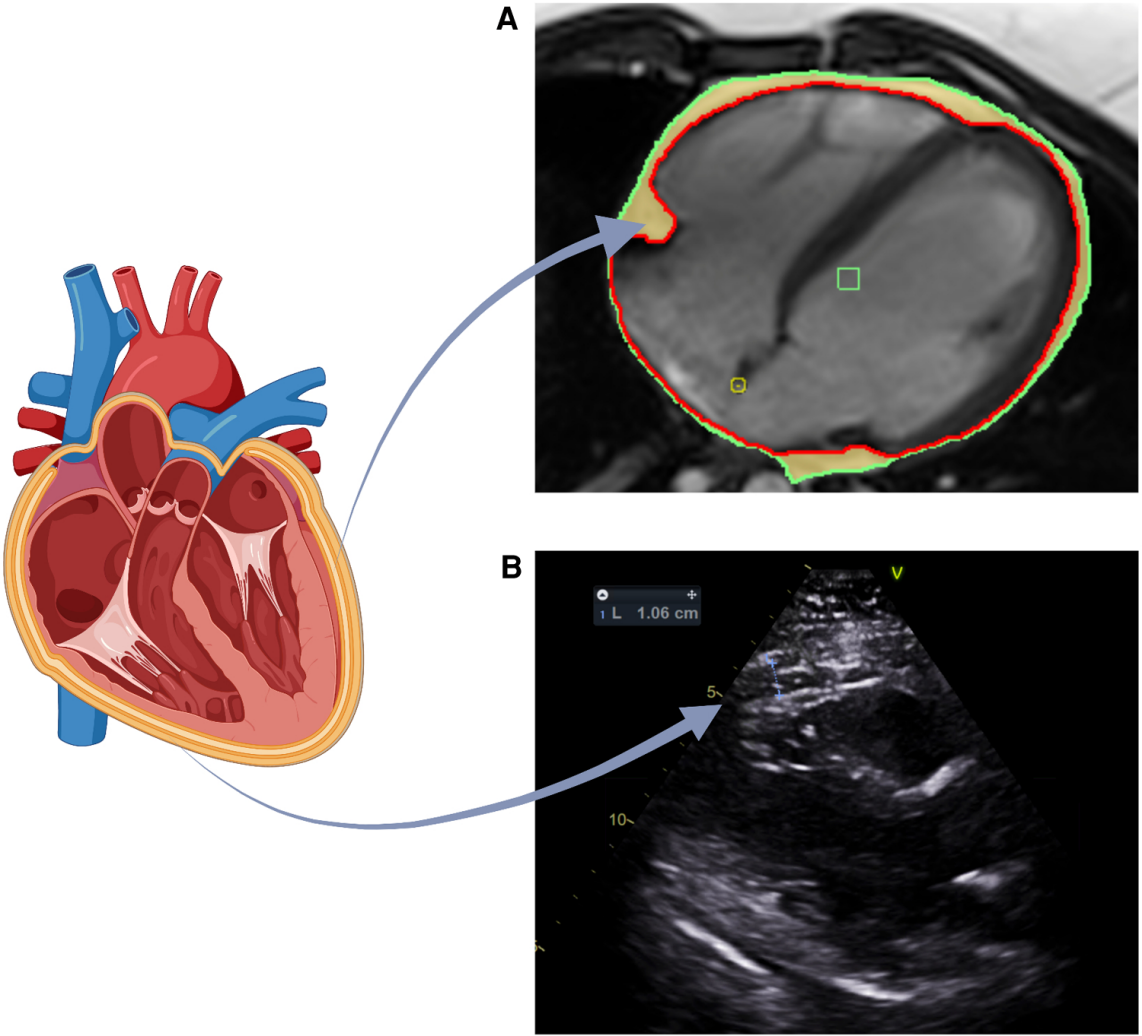
Assessment of EAT utilizing TTE and CMR (**A**) EAT volume assessed by CMR. EAT is defined as the adipose tissue between epicardium and pericardium. Long axis 4-chamber view: red line marks the epicardium, green line marks the pericardium and highlighted in yellow is the EAT. (**B)** EAT volume assessed by TTE. Parasternal long axis view used to measure EAT thickness anterior to the right ventricular free wall. The images shown for each technique were acquired from our institute (St Vincent's Hospital Sydney, Australia) with ethics approval.

TTE has been used for quantification of EAT, which is demonstrated by the echo-lucent area along the right ventricular free wall between the epicardium of the right ventricle and parietal pericardium (57). It is not possible to estimate volumetric EAT nor define specific locations of EAT using TTE due to its limited spatial resolution. Epicardial adipose tissue around the entire heart (global EAT), as well as LA EAT can be easily visualized in CMR (Figure 4) using different techniques, including anatomical chemical shift assessment utilizing the SSFP cine sequence, and more specific sequences that allow for fat quantification using spectroscopic water-fat separation methods such as the VARPRO multipoint Dixon algorithm (58). CMR allows estimation of EAT volume mass as well as assessment of EAT that is localized to particular regions such as the LA. Estimation of EAT using CMR does not require the use of gadolinium-based contrast agent and is radiation-free. Heart rate and rhythm also do not cause any significant impact in image quality to assess EAT using CMR, therefore, it can be used in patients with AF, which is a significant strength of this method.

Volumetric quantification of EAT has several clinical implications. EAT is a risk factor and independent predictor of AF development and recurrence after ablation (55). The location of EAT is important in AF, and regional EAT distribution has emerged as an important factor and potential substrate for the pathogenesis of AF (59). EAT has also been suggested to have a role in heart failure, particularly in patients with HFpEF (60). The volume of EAT is significantly higher in patients with HFpEF than in healthy individuals. The association between EAT and heart failure with reduced ejection fraction remains controversial with conflicting studies to date and remains an area of ongoing research (61).

### 2.5. Sphericity Index

In parallel with the shift in focus from LA diameter to LA volume, there is increasing awareness that LA dilatation occurs in an asymmetric manner, progressing from a discoid shape towards a sphere (62). As a result of this, the sphericity index has been proposed as measure of atrial (or ventricular) geometric remodeling. This shape change is thought to result from progressive increases in intra-atrial pressure and volume. Under the laws of physics, a spherical shape has the most optimal surface:volume ratio, which is the lowest possible surface area required to bound any given volume, and hence can accommodate increasing volume with the least amount of wall stress/tension (63). While this mechanistic explanation is appealing, evidence-based data linking the sphericity index to changes in intra-atrial pressure are lacking.

LA sphericity index has been used as an independent predictor of hospitalization for heart failure in patients with dilated cardiomyopathy (64), but its correlation with atrial fibrillation is still not well understood (65). LA sphericity has been proposed as a potentially earlier and more sensitive indicator of atrial fibrillation-related atrial remodeling compared to traditional markers such as LA enlargement (63). However, a recent study shows that LA sphericity is importantly affected by extrinsic factors such as sex and body length but does not correlate with the presence or type of atrial fibrillation (65). LA sphericity can be measured using 3D echocardiography, CMR or CT.

### 2.6. Pulmonary vein morphology and function

Assessment of pulmonary vein (PV) characteristics can provide incremental information about LA pressure, LA/LV function and atrial fibrillation risk. TTE Doppler assessment of PV flow has been used to obtain the S (systolic), D (diastolic), and the PV reversal velocities, representing reservoir, conduit, and booster pump function, respectively, but successful evaluation requires good quality spectral Doppler tracings. Dilatation of the PV and ostia has been found in patients with paroxysmal atrial fibrillation by angiography and CMR (66–68). In addition, pathological examination has demonstrated the existence of muscle fibers resembling atrial myocardium that are arranged circumferentially around the PV ostia with extension several centimeters down the PV walls (69). These muscle fibers have been shown to impart active contractile function up to 15 mm from the PV ostia on 4D multi-slice CT, with most pronounced contractility observed within the superior PV (70). Tracking of passive or active modulation of PV diameter in conjunction with measurement of PV flow and atrial dynamics can be achieved using SSFP and phase contrast flow-encoded CMR. The inter-relationships between these PV properties and atrial conduit, booster pump, and reservoir function are incompletely understood. In addition to the observed contractility of PV, automaticity and triggered activity originating from the PV is highly prevalent in patients with paroxysmal atrial fibrillation (71). Intravascular ultrasound studies have shown that patients with atrial fibrillation have thicker PV myocardial tissue, with areas of thickening coinciding with abnormal electrograms and PV ectopy (72).

### 2.7. 4D and 5D flow

Evaluation of blood flow velocities using pulsed-wave or continuous-wave Doppler is an integral part of TTE and is commonly used to assess valvular function. Doppler echocardiography interrogates the blood flow velocity component in the direction of the ultrasound beam providing 2D flow analysis. This approach, however, is limited by factors such as variable velocity assessment (due to beam alignment), limited acoustic windows, and operator expertise (73). Further, the calculation of mean velocities and net flow is often based on assumptions regarding the underlying flow profile and vessel cross-sectional area which may result in inaccurate flow quantification in the presence of complex flow and/or vessel geometry. In contrast, intra-cavity LA blood flow patterns are challenging to explore by conventional techniques, especially given the complexities of simultaneous PV inflow. Transesophageal echocardiography can provide information about LA appendage blood flow characteristics and is clinically useful for determining the propensity for blood stasis and thromboembolic risk (74). Transesophageal echocardiography requires esophageal intubation and is not entirely devoid of procedural hazards.

More recently, CMR-based techniques have been refined to include four-dimensional (4D) flow that encodes velocity in all three spatial directions (3D) as well as time. This unique technique enables a wide variety of options for visualization and quantification of flow, ranging from basic aspects such as flow volume and peak velocity to more advanced features such as the estimation of hemodynamic effects at the vessel wall and myocardium, and visualization of flow pathways in the heart and great vessels (75). 4D flow CMR has demonstrated the capacity to provide comprehensive hemodynamic assessment of cardiac chambers and great vessels. It has been used for evaluation of LA flow dynamics such as vortex formation (76), quantification of velocity distribution (77), and estimation of global LA stasis (78). A recent study demonstrates that 4D flow features such as vortex size were associated with the CHA_2_DS_2_-VASc thromboembolic risk score (79). Another advantage of 4D flow CMR is the ability for retrospective placement of analysis planes at any location within the acquisition volume (75). However, data acquisition using 4D flow is time-consuming with scan time dependent on breathing patterns of the subject, limiting the applicability of this method in a clinical setting.

Recent explorations into multi-dimensional and self-gated imaging have pushed the boundaries of conventional cardiac imaging towards five-dimensional (5D) flow framework has been developed to overcome the time-consuming data acquisition needed with 4D flow (Figure 3). This framework features a continuous, free breathing, 3D radial sequence, with interleaved 3D velocity encoding as well as inherent self-gating projections to encode cardiac and respiratory motion without external gating signals (80, 81). This novel technique reduces scan time, which is very important in view of the already long duration of a clinical CMR. It also permits assessment of the major pathophysiologic interactions between the cardiovascular and respiratory systems, which is usually neglected in a single diagnostic testing. 5D flow has been used for atrial function assessment by correlating mean atrial velocities and stasis with atrial fibrillation burden (81).

AF is associated with increased risk of ischemic stroke, attributed to thromboembolism originating in the LA and particularly in the LAA. Changes in LA/LAA hemodynamics (low peak emptying velocities and increased flow stasis) in AF have been associated with thrombus formation and thus stroke risk. However, these studies have primarily employed transesophageal echocardiography, which is semi-invasive, may require sedation, and cannot capture the complex 3D flow dynamics inside the LA and LAA. 5D flow techniques are not technically limited by the irregular rhythm in AF and is a non-invasive technique able to accurately study LA haemodynamics and may potentially be able to assess risk of thromboembolism (81). This applies not only for the atria, but also for the ventricle, which may prove to be useful in patients with not only ACM but also ventricular cardiomyopathy and heart failure.

## 3. Discussion

Understanding of the clinical significance of ACM has been hampered by the failure to recognize this entity and imprecise methods for quantification of atrial structure and function. Both TTE and CMR have been explored, however data that directly compare these techniques are limited. A further issue is the accuracy and reproducibility of the various parameters assessed. In one study, a moderate to poor inter-modality correlation was found between atrial volumes and LAEF (21, 82, 83), with good correlation for atrial reservoir strain (84). More recently, a study of the reproducibility of LA function using CMR demonstrated that LAEF had a better test-retest reproducibility than LA strain, whilst reservoir strain accounted for the most reproducible strain parameter (85).

New imaging techniques for atrial assessment have some limitations. From a technical perspective, there is no universal, standardized, and routinely available atrial-dedicated image analysis software that also incorporates peripheral structures for either TTE or CMR. There is a need for standardization of techniques and establishment of normal reference ranges for the various parameters before clinical application. Furthermore, although the time for image acquisition may be acceptable for clinical use, data analysis for these imaging techniques can be very laborious and take several hours, this is currently a limitation to use this method in clinical practice. In CMR, the development of artificial intelligence techniques has reduced analysis time significantly, but this is not yet available for most atrial analysis techniques. Atrial strain, which is one of the most studied techniques in recent times and has also been included in imaging guidelines (86), is still limited due to measurement variability due to imaging modality, software, and operator factors (87).

A key unresolved question is which parameters are the most sensitive and specific for ACM and most useful for diagnosing this disorder in the clinical setting. Deriving a diagnostic algorithm for ACM will require further comprehensive phenotyping studies of atrial structure and function in various patient cohorts and definition of normal ranges, together with better longitudinal follow-up to evaluate patient outcomes. Ascertaining the causes of ACM is also required in order to understand the genetic and environmental underpinnings of this disorder, some of which may be reversible or prevented.

## 4. Conclusions

ACM is an important clinical entity and potential determinant of heart failure, atrial fibrillation, and thromboembolic risk. The lack of consensus for the diagnosis of ACM and standardization for methods for its assessment have been limiting factors. Advances in imaging methods are providing unprecedented opportunities for comprehensive atrial phenotyping that extends beyond measurement of 2D size to include evaluation of phasic volumes and function, tissue characterization and blood flow. These new and rapidly evolving imaging tools will be instrumental in improving understanding of the causes and consequences of ACM.
